# Supplementary material for: HtrA1 in Gestational Diabetes Mellitus: A Possible Biomarker?
Source: Diagnostics (Basel). 2022 Nov 5;12(11):2705. doi: 10.3390/diagnostics12112705 (PMC9689498; doi:10.3390/diagnostics12112705)
Supplement: Supplementary file 1 [file diagnostics-12-02705-s001.zip › Table S1.pdf]

	ID	Mathernal Age	BMI	Gestational age at delivery	Birth weight	Neonatal gender	Therapy	HtrA1 (ng/ml)
	CTRS5	36	24,98	40,40	3505	1	NO	3,19
	CTRS6	30	21,09	38,20	2800	0	NO	4,30
	CTRS7	36	24,8	41,20	2950	0	NO	0,73
	CTRS8	32	26	39,30	3400	1	NO	1,22
	CTRS9	36	23,93	37,90	3410	1	NO	2,17
	CTRS10	32	23,8	40,90	3260	1	NO	3,90
	CTRS11	29	21,09	40,40	3890	1	NO	3,12
	CTRS13	32	27,75	38,30	3590	0	NO	0,57
	CTRS14	31	24,44	39,40	3350	0	NO	0,74
	CTRS15	27	28,38	40,00	3560	1	NO	0,15
	CTRS16	35	24,96	40,00	3420	0	NO	4,85
	CTRS17	22	18	40,70	3170	1	NO	5,92
	CTRS21	37	28,12	36,40	3105	0	NO	1,04
	CTRS22	34	22	37,60	3530	1	NO	0,78
	CTRS23	27	21,56	40,70	3545	1	NO	4,62
	CTRS24	36	19,48	40,50	3240	1	NO	2,71
	CTRS25	30	29,41	40,80	3965	0	NO	4,64
	CTRS26	40	21,61	37,00	3542	1	NO	1,22
	CTRS27	31	22,47	38,30	3620	0	NO	1,60
CTRL	CTRS28	35	23,73	39,20	3750	0	NO	0,82
	DGS2	45	18,5	39,60	3090	1	Diet	3,42
	DGS3	33	30,11	39,60	4325	0	Diet	8,21
	DGS4	35	21,56	36,00	2735	0	Diet	4,02
	DGS7	36	19,46	39,40	3750	1	Insulin	4,13
	DGS9	36	21,20	38,00	2685	1	Insulin	3,45
	DGS12	33	21,20	37,40	2800	1	Insulin	4,48
	DGS14	35	23,18	33,70	1720	0	Diet	4,75
	DGS15	36	20,19	36,30	2905	0	Diet	6,39
	DGS16	38	21,22	40,40	4035	1	Diet	6,57
	DGS17	36	38	39,30	2515	1	Diet	5,94
	DGS18	35	19,57	39,00	3200	0	Insulin	5,08
	DGS19	30	24,8	40,30	3540	0	Diet	6,19
	DGS20	30	23,8	40,80	3375	0	Diet	4,55
	DGS21	33	21,4	37,20	2505	0	Diet	2,77
	DGS22	47	20,83	38,80	3230	1	Insulin	3,53
	DGS23	44	20,53	38,80	2985	0	Insulin	3,44
	DGS24	39	28,04	39,50	3635	1	Insulin	6,89
	DGS25	38	26,64	38,70	3240	1	Diet	1,85
	DGS27	40	20,55	39,00	3325	0	Diet	1,11
GDM	DGS28	40	35,62	38,70	3390	0	Diet	0,57

Neonatal gender	
female	0
male	1
