# Supplementary material for: HtrA1 in Gestational Diabetes Mellitus: A Possible Biomarker?
Source: Diagnostics (Basel). 2022 Nov 5;12(11):2705. doi: 10.3390/diagnostics12112705 (PMC9689498; doi:10.3390/diagnostics12112705)
Supplement: Supplementary file 1 [file diagnostics-12-02705-s001.zip › Table S2.pdf]

GDM

ID	Maternal age	smoking	Diet	Physical activity	BMI pre-pregnancy	BMI actual	Type of delivery	Gestational age at delivery (weeks)	Neonatal gender	Birth weight (g)	HtrA1 (ng/ml)
**35**	35	0	4	1	19,44	20,06	0	38	1	3100	0,62
**62**	27	0	3	0	39,54	39,54	1	38	1	2990	1,03
**83**	21	1	3	0	26,23	27,78	0	40	1	3640	1,37
**137**	35	0	4	1	23,10	24,17	0	39	1	3890	1,12
**204**	30	0	3	0	20,79	21,13	0	38	1	3030	0,47
**260**	32	0	3	0	30,86	31,25	0	40	1	4250	1,32
**261**	33	2	3	1	23,73	24,92	1	39	0	3030	2,29
**362**	37	0	2	0	24,22	26,17	1	38	0	3800	1,24
**383**	34	0	2	1	20,76	21,45	0	38	1	2630	1,11
**419**	32	0	3	1	24,02	24,74	0	39	0	3150	1,35
**478**	29	0	3	1	30,86	31,99	1	40	0	3310	0,99
**504**	38	0	4	0	27,89	29,00	0	39	0	2850	1,12
**570**	29	0	2	0	20,15	20,48	0	40	1	3840	0,55
**583**	37	0	2	1	18,65	19,54	1	41	0	3100	0,54
**642**	31	0	2	1	20,50	21,16	0	40	0	3810	1,11
**720**	31	0	2	3	21,61	21,97	0	37	1	3180	1,22
**616**	34	2	3	1	21,85	23,72	0	40	1	4010	0,61
**862**	24	2	3	1	23,31	23,67	1	41	1	3522	1,50
**879**	36	0	1	1	29,38	29,38	0	40	0	3560	0,78
**952**	24	0	4	3	24,97	25,81	0	41	0	3460	0,82
**959**	27	2	3	1	20,07	21,11	0	41	0	3050	1,09
**965**	26	0	2	1	35,16	35,16	0	40	0	3960	0,77
**985**	29	0	2	0	29,97	29,97	0	40	1	3430	1,18
**992**	33	1	2	1	22,86	23,95	0	39	1	3490	1,18
**999**	37	0	2	3	22,49	23,18	0	41	1	3910	0,48
**1017**	34	2	2	1	22,49	24,22	1	39	0	3830	1,70
**1027**	29	0	2	1	20,08	21,16	0	40	1	3420	1,64
**1040**	27	0	2	0	30,67	30,12	1	38	1	4010	0,41

Controls

**5**	32	0	5	1	25,15	25,53	1	41	1	4170	1,33
**14**	29	0	3	1	21,55	22,26	0	40	1	3650	2,12
**15**	38	1	4	0	24,34	25,15	0	39	0	2900	1,19
**16**	25	0	4	1	27,73	28,13	0	41	0	3450	2,51
**17**	32	0	4	3	19,92	19,92	0	39	0	2866	1,17
**20**	30	0	3	3	20,88	21,38	0	40	1	3500	2,37
**29**	29	0	4	0	24,22	24,22	0	38	0	2990	2,80
**34**	27	0	4	1	18,75	18,16	0	40	0	3150	1,68
**44**	32	0	3	0	21,30	21,67	1	39	1	3450	1,52
**52**	26	0	3	1	23,03	23,56	0	40	0	3720	1,23
**56**	32	0	4	3	30,11	28,89	0	39	1	2950	1,83
**57**	26	2	3	1	19,65	20,57	0	41	1	3700	1,90
**58**	36	0	3	1	20,80	21,87	0	41	0	3750	1,78
**63**	30	0	4	3	27,78	27,78	0	41	0	3600	0,59
**70**	32	0	3	1	22,50	23,03	0	41	0	3310	2,59
**76**	32	0	3	1	21,51	21,69	0	41	1	3660	2,18
**82**	31	0	4	3	22,07	22,07	0	40	1	3230	1,24
**87**	31	2	4	0	23,74	24,80	0	40	1	4240	2,39
**106**	26	0	5	1	20,06	21,91	0	40	0	3744	0,38
**109**	31	2	2	0	21,63	20,34	0	40	0	3170	1,06
**112**	27	0	3	1	17,63	18,61	0	40	0	3410	0,91
**117**	23	2	4	1	18,94	19,10	0	40	1	2890	1,18
**121**	34	0	4	0	22,23	22,41	0	39	0	3550	0,32
**126**	30	0	3	3	24,49	24,73	0	41	0	3240	0,99
**127**	24	0	4	3	21,64	22,94	1	41	1	3800	0,78
**128**	32	2	3	1	21,06	21,06	0	40	1	3350	0,35
**129**	29	0	4	0	28,41	28,38	0	40	1	3170	0,26
**134**	34	0	3	1	20,07	21,45	1	39	0	3320	0,30
**143**	35	2	4	3	19,63	19,63	0	41	0	3170	0,50
**147**	29	0	3	1	22,53	25,14	0	37	0	2800	0,21
**159**	37	0	4	0	19,07	20,28	0	41	0	3070	0,85
**161**	25	1	4	1	22,68	24,54	0	38	0	3350	0,73
**164**	30	0	4	0	24,34	26,70	0	40	0	4200	1,59
**166**	32	2	2	0	23,62	22,98	0	37	1	2760	1,94
**170**	32	0	2	0	15,67	16,98	1	38	0	2700	1,03
**173**	32	0	4	0	22,32	22,68	1	40	0	3250	0,17
**194**	26	0	2	0	25,39	25,39	0	40	0	3650	0,52
**197**	26	0	3	1	23,44	23,44	1	40	1	3650	0,65
**199**	39	2	4	0	35,38	37,46	1	41	1	3540	0,72
**201**	33	0	5	1	21,45	22,58	0	41	1	3410	0,42
**205**	25	1	3	0	18,83	20,43	1	40	0	3000	0,75
**207**	22	0	2	1	18,42	19,84	1	39	0	3200	1,50
**209**	30	0	2	0	32,39	32,01	0	39	1	3160	1,10
**211**	21	0	3	0	19,95	20,70	0	40	0	3040	0,93
**214**	27	0	3	0	21,09	21,88	0	40	0	2930	0,35
**215**	33	0	3	0	18,62	19,57	0	40	1	3480	0,33
**216**	27	0	3	1	22,98	23,51	0	39	0	2960	0,41
**217**	23	0	3	1	19,71	20,08	0	38	1	3100	0,34
**220**	27	2	1	0	22,86	22,86	0	41	1	4206	0,89
**228**	29	0	3	0	19,83	20,57	0	38	1	2970	0,53
**244**	33	2	3	1	30,12	29,76	1	39	0	3210	1,08
**246**	32	0	4	1	28,91	29,69	0	38	1	3330	0,55
**252**	36	2	3	0	28,23	28,23	0	39	0	3100	0,70
**266**	27	0	2	0	17,50	18,07	0	38	0	3730	0,34
**269**	36	0	4	1	23,81	24,01	1	41	1	3450	0,46
**273**	24	2	3	1	20,80	22,05	0	41	0	3380	0,33
**274**	31	0	3	1	23,21	22,85	0	41	1	3240	0,51
**278**	31	0	4	1	20,70	20,70	1	39	0	3290	0,12
**279**	31	2	2	1	22,94	24,13	0	37	1	2550	0,05
**280**	20	0	4	1	20,57	22,04	0	39	0	3350	0,39
**282**	34	0	3	1	20,55	23,03	0	41	1	3480	0,11
**283**	34	0	3	1	23,59	23,59	0	41	1	3490	0,61
**295**	37	0	3	0	22,59	23,31	1	38	1	4000	0,45
**296**	33	0	4	1	18,00	18,73	0	40	1	3690	1,24
**298**	26	0	3	0	21,63	22,65	0	41	1	3770	0,83
**304**	35	0	4	0	24,80	25,33	1	41	1	4070	0,38
**308**	33	2	3	0	22,76	24,30	0	36	1	2555	0,62
**310**	34	2	3	1	17,58	17,97	0	41	0	3080	0,58
**312**	30	0	2	1	19,72	20,55	1	40	1	3410	0,65
**315**	35	0	2	1	18,87	19,60	1	41	0	2960	0,01
**321**	29	0	4	1	17,43	17,43	1	38	1	3250	0,18
**323**	33	0	4	1	20,06	20,99	0	40	1	3830	0,34
**327**	32	1	2	0	19,53	20,70	0	35	1	2754	0,68
**336**	20	1	2	0	19,23	19,96	1	41	0	3700	0,42
**341**	31	0	3	1	19,61	20,11	0	41	1	3140	0,44
**346**	39	0	3	1	21,30	21,85	0	41	0	3980	0,26
**354**	31	0	2	1	18,52	18,90	0	39	0	3550	0,25
**363**	32	0	3	1	21,11	21,45	1	40	0	3860	0,10
**366**	37	0	3	1	18,73	19,47	0	38	1	3500	0,59
**368**	28	0	2	1	25,95	26,30	0	40	0	3980	0,15
**369**	31	0	3	1	18,17	18,69	1	38	0	2700	0,22
**370**	18	1	3	0	15,43	16,16	0	40	0	3370	0,93
**378**	32	0	3	1	23,67	23,92	1	40	0	3570	0,30
**381**	35	0	4	1	23,24	23,24	0	40	0	3484	0,18
**396**	33	0	4	1	18,83	19,43	0	41	0	3130	0,53
**398**	26	1	1	1	19,31	19,31	0	39	1	2628	0,39
**399**	33	0	3	0	23,93	22,86	0	39	1	3980	0,30
**405**	29	0	2	1	19,75	20,68	0	40	0	4000	0,22
**406**	28	1	3	1	21,38	22,05	0	40	0	2940	0,44
**411**	38	0	3	1	22,43	22,43	1	40	1	3500	0,15
**414**	38	0	2	1	29,39	32,00	1	38	0	3550	1,28
**415**	34	0	4	1	22,74	23,02	0	41	1	3440	0,40
**420**	25	0	3	0	19,47	19,83	0	40	1	3200	0,35
**421**	31	0	3	1	21,30	21,67	0	40	0	3400	0,45
**422**	36	2	2	1	22,66	23,83	0	40	1	4430	0,84
**424**	27	0	4	1	21,12	21,36	0	40	0	3170	0,43
**425**	38	0	3	0	20,76	21,80	0	38	1	3650	0,18
**428**	28	0	2	0	25,71	25,71	1	38	1	2900	0,66
**435**	33	0	4	1	24,14	24,97	1	39	0	2780	0,54
**440**	31	0	4	1	18,29	19,36	0	40	0	2930	0,41
**442**	26	2	2	1	19,03	19,72	0	39	1	3480	0,60
**443**	35	0	2	0	20,88	21,55	0	40	1	4030	0,64
**447**	31	0	3	1	22,05	22,39	0	40	1	3910	0,49
**450**	35	0	3	1	23,66	23,66	0	40	0	3820	0,70
**454**	28	0	3	1	20,57	21,22	1	40	0	2890	0,65
**461**	30	0	3	0	18,00	19,10	0	40	1	3370	0,32
**465**	37	0	3	0	20,02	20,44	1	40	1	2670	0,27
**466**	29	2	2	0	22,72	23,05	0	41	0	3000	0,57
**468**	27	0	3	0	25,39	27,34	1	40	0	3490	0,83
**474**	32	0	2	0	20,28	20,69	0	39	0	3340	0,29
**475**	27	0	4	1	20,96	21,72	0	39	1	3380	0,36
**481**	22	0	2	0	23,42	24,54	0	40	0	3870	2,04
**484**	34	0	1	0	23,61	24,88	0	40	1	2860	0,58
**488**	26	0	3	1	25,65	26,03	1	39	0	3100	0,44
**494**	39	0	2	0	25,77	26,85	0	41	1	3740	0,78
**496**	33	0	4	1	21,61	21,61	0	40	0	2840	0,75
**502**	32	0	4	0	19,68	20,83	0	39	1	3680	0,28
**503**	32	0	4	1	20,90	21,97	0	40	1	3000	0,15
**512**	27	2	3	0	21,51	21,51	1	38	1	3775	0,92
**513**	26	0	2	1	24,73	24,73	0	38	1	3170	0,37
**514**	31	0	2	0	21,63	21,63	0	36	1	3420	0,69
**515**	28	0	4	1	27,89	28,30	0	40	0	3470	0,74
**518**	31	0	2	0	20,63	20,82	0	41	1	3040	0,40
**521**	35	0	4	3	23,83	25,07	0	40	0	3600	0,39
**523**	36	0	2	1	19,72	18,69	1	39	0	3550	0,20
**524**	37	2	2	1	20,31	20,86	0	38	0	3380	0,45
**531**	29	0	1	1	22,31	22,87	0	38	1	3430	0,76
**537**	35	0	3	1	28,37	28,72	0	40	1	3830	1,25
**542**	32	2	3	1	19,92	20,12	1	39	1	3090	1,05
**546**	24	2	3	0	24,01	24,39	0	38	0	2830	0,46
**547**	30	0	4	1	21,45	21,80	0	38	1	3170	0,25
**549**	39	0	3	0	32,42	31,25	0	38	1	3350	1,13
**550**	30	0	3	1	19,84	20,55	0	39	0	3430	0,49
**552**	34	2	3	1	21,56	22,49	0	39	0	3000	1,24
**553**	34	2	2	0	26,03	25,28	0	40	1	3000	0,74
**557**	22	1	2	0	20,10	21,80	1	38	1	2980	0,60
**559**	29	0	4	1	48,29	48,83	0	40	1	3300	0,62
**564**	30	1	4	3	29,98	29,98	1	38	1	3770	1,01
**568**	31	0	3	1	17,96	17,63	0	38	0	3660	0,90
**571**	19	1	2	0	19,05	19,81	0	38	1	3100	1,31
**573**	32	0	5	1	20,70	20,70	0	41	1	3360	0,44
**574**	35	0	2	1	22,41	22,77	0	39	0	3770	1,79
**582**	31	0	3	1	18,34	18,86	0	41	0	3170	0,87

Smoking	
Not	0
Yes	1
ex	2

Diet	
very poor	0
poor	1
mediocre	2
mean	3
good	4
very good	5

Physical activity	
Walking (min 30 min) / moderate physical activity (e.g specific courses for pregnant women)	
yes / yes	3
yes/ not	1
not /yes	1
not/ not	0

Type of delivery	
Vaginal	0
C-section	1

Neonatal gender	
female	0
male	1

**584**	27	0	4	1	20,76	20,76	0	40	1	3280	0,56
**586**	26	2	2	0	18,44	18,82	0	41	0	3140	0,77
**597**	34	0	2	0	18,37	18,73	0	35	1	2925	0,66
**599**	41	0	2	1	20,32	21,38	1	40	1	3470	0,96
**602**	38	0	3	1	23,32	24,00	0	41	1	3350	1,43
**613**	28	0	3	0	30,49	30,49	1	41	1	3870	0,66
**615**	30	2	3	0	18,69	19,90	0	40	0	3420	0,81
**623**	35	0	2	0	19,72	20,13	1	37	1	2640	0,88
**627**	29	0	4	1	25,39	27,34	0	38	1	3670	1,26
**628**	32	0	4	3	20,03	20,63	1	39	0	3225	0,89
**633**	29	1	1	1	18,22	19,52	0	39	1	3280	1,20
**634**	33	2	2	1	19,63	20,43	0	40	1	2890	0,62
**635**	32	0	3	1	20,70	21,09	1	40	1	3730	0,36
**636**	31	0	4	1	20,20	20,57	0	39	1	3350	0,95
**640**	37	0	3	3	18,74	20,78	0	37	1	3050	0,74
**643**	30	2	3	1	21,26	22,32	0	41	1	3850	0,79
**647**	28	0	2	0	32,91	32,91	0	41	1	3540	0,63
**651**	30	0	3	0	24,97	25,81	0	40	0	3300	1,93
**657**	32	2	2	1	18,43	19,23	0	40	0	3118	0,70
**776**	32	0	3	1	24,84	24,00	0	40	1	3560	0,82
**826**	34	0	4	0	21,48	21,48	0	40	1	3620	0,40
**827**	22	1	3	0	29,17	29,55	1	40	0	3020	0,56
**830**	40	0	5	3	18,73	19,47	0	40	0	3430	0,42
**850**	37	0	4	0	22,76	22,95	0	37	0	2610	0,41
**851**	28	2	2	1	19,13	20,37	0	41	0	3040	1,34
**854**	34	0	3	3	19,59	19,92	0	39	0	3300	0,43
**857**	34	0	2	1	20,32	21,45	0	40	1	4090	0,52
**861**	40	0	2	1	22,96	22,77	0	40	0	3060	0,87
**863**	36	0	2	1	20,32	20,89	0	39	1	3240	0,41
**865**	29	0	2	0	18,65	19,72	0	38	1	3060	1,53
**868**	32	0	2	1	32,87	33,91	0	39	1	3720	0,62
**873**	35	0	3	1	20,70	21,88	0	40	1	3050	1,68
**876**	32	0	2	1	23,71	24,09	0	41	0	3330	1,13
**877**	23	2	2	1	30,10	30,80	0	41	0	3460	0,73
**878**	27	0	1	1	23,24	24,39	0	40	1	3300	0,75
**880**	27	2	2	1	19,62	21,03	0	40	1	3460	0,76
**882**	36	2	1	1	20,68	20,86	0	39	1	3250	0,41
**888**	28	0	2	0	20,81	20,81	0	39	1	3330	0,99
**896**	26	0	2	1	21,80	22,49	0	41	0	3110	1,22
**905**	27	2	2	3	17,58	18,75	0	39	1	3800	0,63
**906**	35	0	3	1	20,76	20,07	0	40	1	3680	1,26
**910**	33	0	2	0	24,17	23,05	0	39	1	3610	0,55
**914**	29	0	2	1	18,29	19,00	0	39	1	3180	0,88
**920**	34	0	3	1	25,95	25,30	0	40	0	3170	1,63
**934**	33	0	3	1	21,21	21,64	0	39	1	2910	0,26
**938**	35	0	3	0	20,70	21,64	1	40	0	3170	0,65
**939**	27	0	3	1	22,59	22,77	0	39	1	2820	0,29
**942**	36	2	3	1	20,35	21,89	0	38	0	2550	1,47
**945**	27	2	2	1	21,30	22,31	0	39	1	3300	1,09
**955**	31	0	3	1	20,08	21,56	0	39	0	3320	0,57
**973**	35	0	2	1	20,39	20,94	0	39	1	3280	0,52
**974**	38	2	2	1	27,68	28,37	0	41	0	2945	0,49
**976**	28	0	2	0	18,07	18,07	0	39	1	3220	0,57
**982**	36	0	2	1	21,94	22,68	0	40	1	3640	0,29
**983**	26	2	2	1	21,53	21,53	0	38	0	3420	0,61
**991**	28	0	2	1	18,94	18,25	0	40	1	3510	1,27
**993**	27	0	2	1	24,09	25,51	0	39	1	3460	1,82
**995**	25	0	3	1	18,73	19,65	0	41	1	2955	1,02
**1004**	33	2	2	1	19,53	19,53	0	41	0	3130	1,40
**1005**	27	0	2	0	18,90	19,68	0	39	1	3680	1,30
**1006**	31	0	3	1	24,97	24,56	0	41	1	3055	0,73
**1007**	28	0	2	1	21,41	24,68	0	39	0	3150	0,80
**1009**	30	2	3	1	20,05	20,05	0	41	1	4250	0,52
**1010**	29	0	4	1	24,27	24,80	0	41	0	3900	0,95
**1016**	30	2	4	1	21,05	21,72	0	40	1	3500	1,11
**1020**	31	0	2	0	23,39	23,39	0	41	0	3570	0,91
**1025**	29	2	3	0	18,63	19,01	0	39	1	3325	1,03
